# Supplementary material for: Arrhythmia and Heart Rate Variability during Long Interdialytic Periods in Patients on Maintenance Hemodialysis: Prospective Observational Cohort Study
Source: J Clin Med. 2022 Dec 29;12(1):265. doi: 10.3390/jcm12010265 (PMC9820857; doi:10.3390/jcm12010265)
Supplement: Supplementary file 1 [file jcm-12-00265-s001.zip › jcm-2063600-supplementary.pdf]

Table S1. HRV parameter changes in patients with and without significant arrhythmias

	Without significant arrhythmia		Significant arrhythmia	
	**Mean change per day**	***p*-value**	**Mean change per day**	***p*-value**
**SDNN**	-0.003 (-0.029–0.022)	0.793	0.032 (0.007–0.057)	0.013
**SDANN**	0.021 (-0.003–0.044)	0.084	0.044 (0.022–0.067)	<0.001
**RMSSD**	0.146 (0.113–0.178)	<0.001	0.053 (0.025–0.082)	<0.001
**pNN50**	0.290 (0.209–0.372)	<0.001	0.060 (-0.017–0.137)	0.124
**VLF**	0.083 (0.053–0.113)	<0.001	0.028 (0.001–0.056)	0.042
**LF**	0.044 (0.024–0.064)	<0.001	0.001 (-0.019–0.021)	0.922
**HF**	0.078 (0.059–0.098)	<0.001	0.012 (-0.005–0.028)	0.161
**LF/HF ratio**	-0.118 (-0.177–-0.059)	<0.001	-0.015 (-0.074–0.043)	0.605

The statistical results were acquired from linear mixed models using Satterthwaite approximations for the degrees of freedom. The mean changes in HRV parameters per day are presented as estimates with 95% confidence intervals. SDNN, SDANN, RMSSD, pNN50, and the LF/HF ratio values were natural log-transformed, and the VLF, LF, and HF values were square root-transformed. SA, significant arrhythmia; SE, standard error; SDNN, standard deviation (SD) of normal-to-normal intervals during an hour; SDANN, SD of the averages of the NN intervals in all five-minute segments during an hour; RMSSD, square root of the mean square of the differences between adjacent NN intervals; pNN50, percentage of the number of pairs of adjacent NN R-R intervals differing by > 50 ms in the total NN intervals; VLF, very-low-frequency (0.003–0.04 Hz) power; LF, low-frequency (0.04–0.15 Hz) power; HF, high-frequency (0.15–0.40 Hz) power.

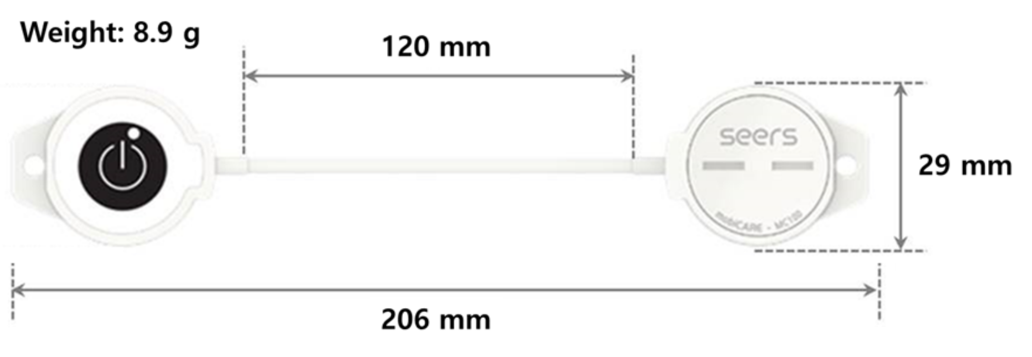

Product serial number	mobiCARE-MC100
Size	Width 29 mm, Length 120 mm
Weight	8.9 g
Sensors	Electrodes, 3-axis accelerometers, 3-axis gyroscopes
Sampling rate	256 Hz
Operating time	At least 72 hours

Figure S1. Image and detailed specifications of the patch-type ECG monitoring device.
